# Deletion of the calcium-binding protein S100β alters axon initial segment development in the visual cortex

**DOI:** 10.3389/fncel.2026.1895033

**Published:** 2026-07-27

**Authors:** Yanis Inglebert, Rafael Sanz-Gálvez, Danna Quetzali Chavez Lorenzana, Eva Lafaverges, Arlette Kolta

**Affiliations:** 1 Department of Neurosciences, Université de Montréal, Montréal, QC, Canada; 2 Centre Interdisciplinaire de Recherche sur le cerveau et l’apprentissage (CIRCA), Montréal, QC, Canada; 3 Faculté de Médecine Dentaire, Université de Montréal, Montréal, QC, Canada

**Keywords:** astrocytes, axon initial segment (AIS), development, S100β, visual cortex

## Abstract

The axon initial segment (AIS) is a key structure for fine-tuning neuronal excitability. It plays a particularly important role during development across many sensory systems, where it contributes to network refinement and the adjustment of excitability levels. In the visual cortex, the AIS undergoes a significant period of plasticity that helps adjust neuronal excitability during key developmental stages such as eye opening and the onset of binocular vision. This development is tightly regulated by numerous factors, including glial cells. The calcium-binding protein S100β, which is primarily expressed in astrocytes, has previously been implicated in axonal development. We therefore asked whether it might also contribute to the normal development of the AIS in the visual cortex. Here, we characterize the consequences of S100β loss on AIS development and examine its impact on neuronal excitability.

## Introduction

The axon initial segment (AIS) is a specialized structure located near the soma of most excitatory neurons, enriched in ion channels and responsible for action potential initiation (Fréal and Hoogenraad, 2025). Its geometry strongly regulates neuronal excitability: a longer AIS generally increases excitability, whereas a shorter AIS reduces it (Grubb and Burrone, 2010; Fréal et al., 2023). Accordingly, structural plasticity of the AIS represents a powerful mechanism for tuning neuronal excitability across multiple timescales. AIS structure is dynamically regulated during postnatal development across sensory systems. In mouse visual cortex neurons, AIS length undergoes a three-phase maturation process characterized by an initial elongation from P7 to a peak around P10-P15 near eye opening, followed by a marked shortening beginning around P21 and reaching a minimum at ∼P28 during the critical period of ocular dominance plasticity, and a moderate re-elongation into adulthood (Gutzmann et al., 2014). This developmental remodeling has also been reported in other sensory systems, including the somatosensory and auditory systems (Akter et al., 2020; Jamann et al., 2021). Notably, across these systems the period at which the AIS reaches its maximal length coincides with the onset of sensory function, such as eye opening, the onset of hearing, or the beginning of active environmental exploration. However, the molecular mechanisms regulating developmental AIS remodeling remain incompletely understood. Astrocytes play a critical role in brain development, acting as key regulators of the formation, maturation, and refinement of neural circuits (Ribot et al., 2021; Foubert et al., 2023; Kim and Chung, 2023) and have been shown to control critical-period closure in the mouse visual cortex, (Ribot et al., 2021). They release multiple gliotransmitters, including S100β, a multifunctional calcium-binding protein implicated in several aspects of neurodevelopment, such as glial differentiation and maturation, neurite outgrowth, and axonal growth regulation (Donato et al., 2009; Hernández-Ortega et al., 2024). The effects of S100β are both intra- and extracellular in nature. Intracellularly, it regulates calcium signaling and homeostasis, while extracellularly it binds directly to the multiligand Receptor for Advanced Glycation End-products (RAGE), triggering downstream cascades such as the ERK1/2 and Protein Kinase B pathways (Donato, 2007). Given its established role in axonal development, we hypothesized that loss of S100β would disrupt normal AIS maturation. It should be noted, however, that the constitutive knockout model employed here precludes direct assessment of cell-type-specific contributions or interactions with peripheral organs, as S100β expression is not restricted to the brain. Therefore, we compared AIS length in layer V pyramidal neurons (L5PNs) of the visual cortex between WT and S100β-KO mice during development using immunohistochemistry against the anchoring protein Ankyrin-G (Ank-G) as a marker of the AIS. Whole cell patch-clamp recordings were also used to assess excitability changes.

## Results

### Loss of S100β disrupts normal axon initial segment development in the visual cortex

**FIGURE 1:**
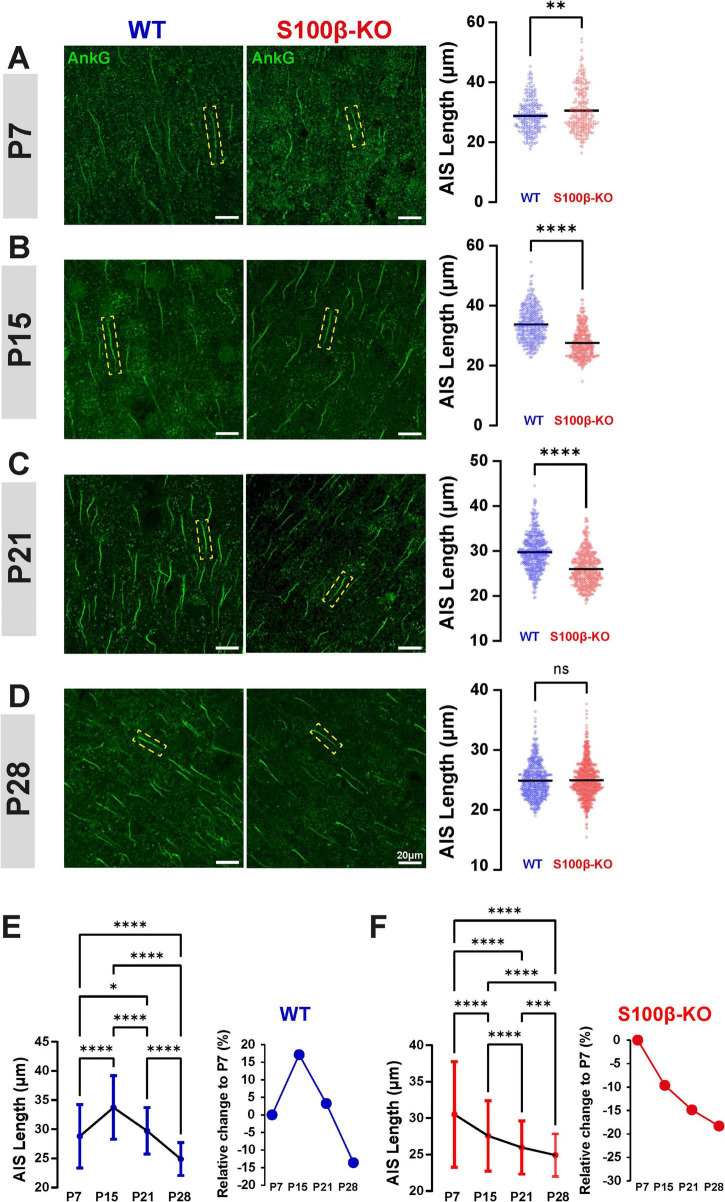
Loss of S100β disrupts normal development of the axon initial segment (AIS). **(A–D)** Left: Ankyrin-G (ankG, green) immunolabeling at successive developmental stages (P7, P15, P21 and P28) in layer 5 of the mouse visual cortex. Right: Quantification of AIS length in wild-type (WT, blue) and S100β knockout (S100β-KO, red) mice; black bars indicate the mean. WT P7 (*n* = 265 from 4 mice across 12 sections); KO P7 (*n* = 257 from 5 mice across 10 sections); WT P15 (*n* = 368 from 4 mice across 13 sections); KO P15 (*n* = 393 from 4 mice across 11 sections); WT P21 (*n* = 477 from 4 mice across 16 sections); KO P21 (*n* = 502 from 3 mice across 12 sections); WT P28 (*n* = 844 from 5 mice across 21 sections); KO P28 (*n* = 554 from 5 mice across 25 sections); **(E)** Developmental changes in AIS length in WT mice expressed as absolute values and as relative changes normalized to the P7 measurement. **(F)** Developmental changes in AIS length in S100β-KO mice, expressed as absolute values and as relative changes normalized to the P7 measurement. Note the marked reduction in AIS length relative change in S100β-KO mice compared with WT controls. Data are presented as mean ± S.D. Statistical significance was set at *P < 0.05, **P < 0.01, ***P < 0.001, and ****P < 0.0001; NS indicates not significant.

To investigate the involvement of the S100β protein in the temporal development of the AIS in the visual cortex, we first examined the normal maturation of the AIS in L5PNs of the visual cortex in WT mice at four developmental time points: P7, P15, P21, and P28 as depicted in Figures 1A–D. Consistent with previous findings (Gutzmann et al., 2014), AIS length underwent two distinct developmental phases. An initial elongation phase spanned from P7 (28.80 μm ± 0.33) to P15 (33.72 μm ± 0.27), followed by a progressive and significant shortening phase extending from P15 through P21 (29.67 μm ± 0.19) and P28 (24.82 μm ± 0.12) (Figure 1E). In contrast, S100β-KO mice displayed a continuous and progressive reduction in AIS length (Figure 1F) across all timepoints examined, declining steadily from P7 (30.51 μm ± 0.43) through P15 (27.07 μm ± 0.25), P21 (26.15 μm ± 0.17), and P28 (24.92 μm ± 0.1), with no initial elongation phase observed. In addition, the magnitude of AIS shortening across development was markedly impacted in S100β-KO mice compared to WT. In WT mice, AIS length first increased by 17.1% from P7 to P15, followed by a gradual reduction of 13.57% between P15 and P28 (Figure 1E, Right). In contrast, S100β-KO mice displayed a progressive and uninterrupted length reduction of 18.32% from P7 to P28, with no initial elongation phase (Figure 1F, Right). In addition, a clear difference in AIS length can be observed between WT and S100β-KO at P7 (*P* = 0.002, 28.80 μm ± 0.33 vs. 30.51 μm ± 0.43, Figure 1A), P15 (*P* < 0.0001, 33.72 μm ± 0.27 vs. 27.07 μm ± 0.25, Figure 1B) and P21 (*P* < 0.0001, 29.67 μm ± 0.19 vs. 26.15 μm ± 0.17, Figure 1C) while no difference persisted at P28 (n.s, 24.82 μm ± 0.12 vs. 24.92 μm ± 0.1, Figure 1D). These results indicate that the loss of S100β disrupts the normal development of the AIS in the visual cortex.

### Loss of S100β resulted in reduced excitability

**FIGURE 2:**
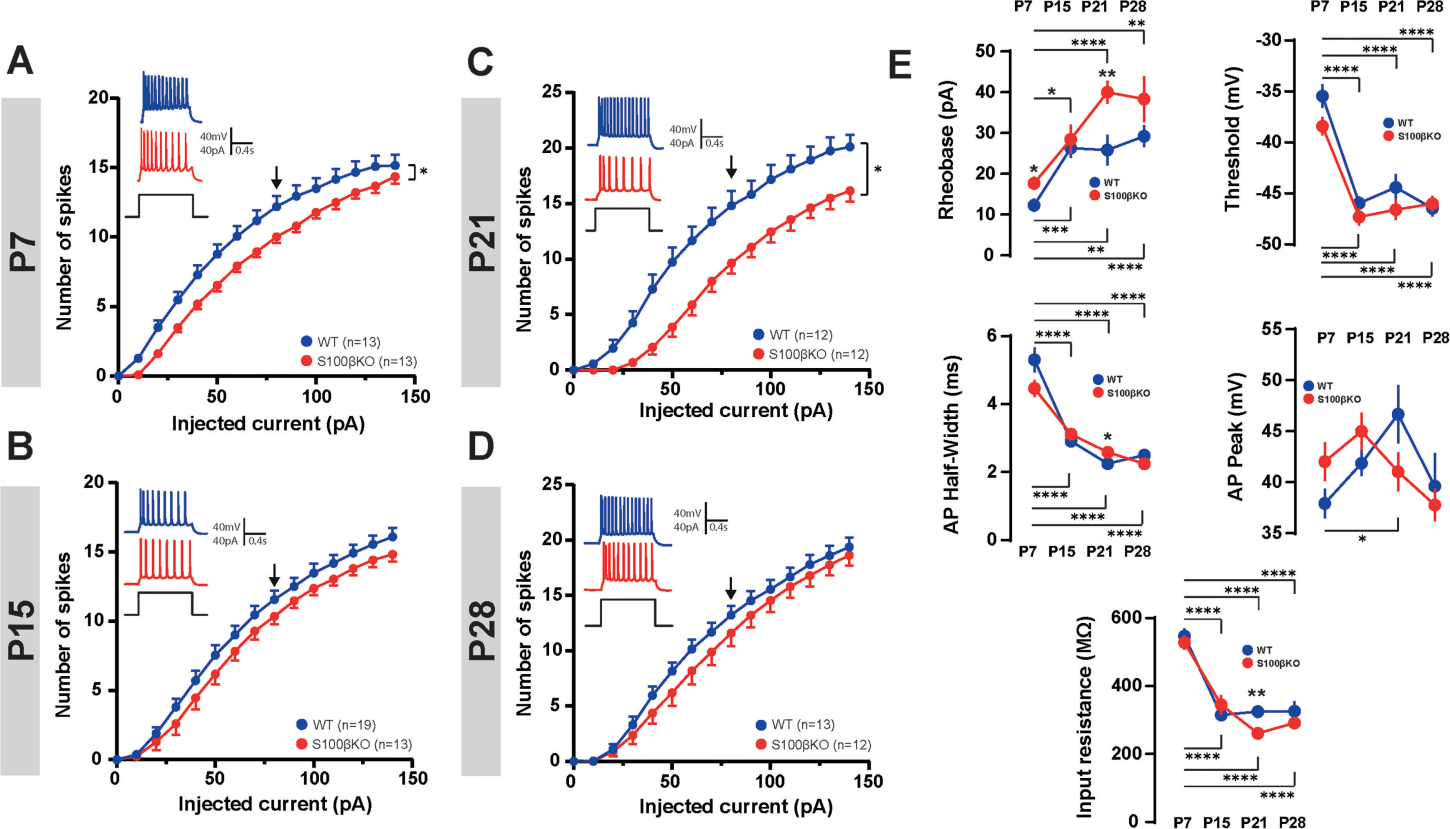
Developmental electrophysiological profile between WT and S100β-KO mice. **(A–E)** Left: Input–output relationships (0–150 pA, Δ = 10 pA) at different developmental stages (P7, P15, P21, and P28) in WT (blue) and S100β-KO (red) mice. Insets: Show typical membrane responses elicited by injection of current pulses corresponding to the black arrow on the curves. Right: Electrophysiological parameters (action potential half-width, AP peak amplitude, spike threshold, and rheobase) measured at the corresponding developmental time points in WT (blue) and S100β-KO (red) mice. Black bars indicate median values. WT P7 (*n* = 13 from 2 mice); KO P7 (*n* = 13 from 2 mice); WT P15 (*n* = 19 from 2 mice); KO P15 (*n* = 13 from 2 mice); WT P21 (*n* = 12 from 3 mice); KO P21 (*n* = 12 from 2 mice); WT P28 (*n* = 13 from 3 mice); KO P28 (*n* = 12 from 2 mice); Statistical significance was set at *P < 0.05, **P < 0.01, ***P < 0.001, and ****P < 0.0001. Data are expressed as mean ± SEM.

To determine whether the developmental differences in AIS length observed between WT and S100β-KO mice are associated with altered neuronal excitability, we performed electrophysiological recordings at P7, P15, P21, and P28 and generated input–output (I/O) curves (0–140 pA, Δ = 10 pA) in presence of synaptic blockers. Other electrophysiological parameters (Table 1) associated with excitability, including action potential half-width, peak, threshold, rheobase and input resistance were also analyzed. Changes in AIS length observed in WT animals would suggest a rise in excitability from P7 to P15, followed by a gradual and sustained decline through P28. Unexpectedly, however, no significant difference in excitability is detected between P7 and P15 in WT animals. Instead, excitability increases significantly from P7 onward, with differences emerging at P21 and persisting at P28 (P7 vs. P21: Two-way RM ANOVA, F(14, 322) = 13.38, *P* < 0.0001; P7 vs. P28: Two-way RM ANOVA, F(14, 336) = 19.61, *P* < 0.0001; P15 vs. P21: Two-way REML ANOVA, F(14, 419) = 10.58, *P* < 0.0001; P15 vs. P28: Two-way REML ANOVA, F(14, 405) = 9.13, *P* < 0.0001). In S100β-KO mice, by contrast, a significant increase in excitability is observed only between P28 and the earlier time points P7 and P15 (P7 vs. P28: Two-way RM ANOVA, F(14, 322) = 12.14, *P* < 0.0001; P15 vs. P28: Two-way RM ANOVA, F(14, 322) = 5.203, *P* < 0.0001). Comparisons of input/output (I/O) curves across developmental stages in WT animals thus reveal significant differences in excitability between postnatal ages, though these do not necessarily mirror changes in AIS length. This dissociation likely reflects the contribution of other parameters that influence neuronal excitability, such as input resistance, which is markedly elevated at P7. As with the other parameters examined, input resistance undergoes its most pronounced change between P7 and P15 (Figure 2E), with values stabilizing thereafter through P28 (Figure 2E). Despite showing a very similar developmental profile for most parameters (Figure 2E), S100β-KO showed generally lower excitability than WT animals, when comparing their I/O curves. These differences were modest and non-significant at P15 and P28 (Figures 2B, D), but were more pronounced at P7 and P21, where S100β-KO mice exhibited a clear downward shift in the I/O curve, indicating a significant reduction (P21: Two-way RM ANOVA, *F*(15, 330) = 8,960, *P* < 0.0001; P7: Two-way RM ANOVA, *F*(14,336) = 2,917, *P* = 0.0003) in neuronal excitability (Figure 2A, C). Similarly to I/O curves, no significant differences were observed in all parameters at P15 or P28 (Figure 2E). At P21, however, both the rheobase (*P* < 0.01, 25.83 pA ± 3.78 pA vs. 40 ± 2.75) and the half-width (*P* < 0.05, 2.24 ms ± 0.1 vs. 2.58 ms ± 0.11) were significantly increased in S100β-KO mice, and the input resistance significantly decreased (*P* < 0.01, 324.7 ± 18.9 vs. 260.7 ± 9.42 MΩ) whereas the other parameters showed no differences (Figure 2E). At P7, only a significant reduction of the rheobase could be observed (*P* = 0.03, 12.3 pA ± 1.66 pA vs. 17.7 pA ± 1.66).

**TABLE 1:** Electrophysiological characteristics of L5PNs of the visual cortex from WT and S100β-KO animals.

Developmental stage	Spike threshold (mV)	AP peak (mV)	AP half-width (ms)	Rheobase (pA)	Input resistance (MΩ)
P7 – WT	−35.46 ± 1.13	37.92 ± 1.47	5.3 ± 0.37	12.3 ± 1.66	546,86 ± 1.66
P15 – WT	−45.96 ± 0.67	41.85 ± 1.25	2.90 ± 0.11	26.31 ± 2.32	314.51 ± 14.31
P21 – WT	−44.41 ± 1.29	46.66 ± 2.86	2.24 ± 0.1	25.83 ± 3.78	324.75 ± 18.84
P28 – WT	−46.45 ± 0.80	39.62 ± 3.25	2.49 ± 0.11	29.23 ± 2.65	325.94 ± 28.9
P7 - S100β-KO	−38.40 ± 0.9	42.03 ± 1.88	4.46 ± 0.25	17.7 ± 1.66	527.83 ± 20.55
P15 – S100β-KO	−47.30 ± 0.85	45.00 ± 1.82	3.11 ± 0.13	28.46 ± 3.55	344.80 ± 28.06
P21 – S100β-KO	−46.59 ± 0.99	41.03 ± 1.90	2.58 ± 0.11	40 ± 2.75	260.68 ± 9.42
P28 – S100β-KO	−46.00 ± 0.75	37.78 ± 1.60	2.243 ± 0.10	38.33 ± 5.62	291.14 ± 16.77

Values are mean ± SEM. KO, knock-out; WT, wild-type; AP, action potential.

## Discussion

Previous studies have shown that AIS structure is highly dynamic during development in sensory systems such as the visual, auditory, and somatosensory systems. All have shown that this maturation process is particularly sensitive, as sensory deprivation disrupts its normal development (Akter et al., 2020; Jamann et al., 2021; Gutzmann et al., 2014). Understanding the mechanisms governing AIS remodeling during development remains a fundamental question that is still not well understood. Here we show that loss of S100β alters AIS development in layer V of the visual cortex and reduces intrinsic excitability of pyramidal neurons during key stages of maturation.

### S100β contributes to axon initial segment remodeling during postnatal development

We analyzed AIS length at four critical ages for AIS maturation: P7, P15, when AIS length is maximal shortly after eye opening (P13–14); P21, when AIS shortening becomes significant and coincides with onset of the ocular dominance critical period (CP); and P28, when AIS shortening is maximal and coincides with the peak of the CP (Fagiolini et al., 1994; Gordon and Stryker, 1996; Fagiolini and Hensch, 2000). WT mice displayed the previously described pattern (Gutzmann et al., 2014), whereas constitutive deletion of S100β resulted in weaker developmental AIS remodeling. In S100β-KO mice, AIS length changes between P15, P21, and P28 is smaller than in WT animals, suggesting reduced adaptive adjustment of AIS structure during this developmental window. In addition, no AIS increase can be observed between P7 and P15. Consequently, AIS maturation may be delayed, leaving neurons in a relatively immature state when sensory inputs rapidly increase after eye opening and at CP onset. Similar functional immaturity has been reported in visual cortical neurons after dark rearing (Fagiolini et al., 1994; Gutzmann et al., 2014). By P28 we did not observe differences, suggesting that the visual system of S100β-deficient mice eventually converges toward a WT-like state, likely through compensatory mechanisms that remain unknown. During the precritical period, between eye opening (P13–14) and CP onset (P21), some aspects of visual circuits mature independently of visual experience (Crowley and Katz, 1999; White et al., 2001), whereas visual input becomes necessary during the CP to maintain circuit organization (Crair et al., 1998). Within these two stages of functional maturation, our observations appear more consistent with the precritical period, where S100β may participate in intrinsic programmed mechanisms regulating AIS remodeling. Importantly, the CP is not simply an age-dependent maturational process but a cascade of controlled events. Its onset can be delayed (Fagiolini and Hensch, 2000) or accelerated (Huang et al., 1999) depending on cortical manipulation. However, what controls CP onset and the underlying plasticity remains unclear, although evidence suggests correlation-based rules for synaptic reorganization (Domenici et al., 1992; Fagiolini et al., 1994; Gordon and Stryker, 1996), that is, the temporal (Hebbian) correlation between inputs from the two eyes (Song et al., 2000; Smith and Trachtenberg, 2007). When this temporal correlation is disrupted, visual circuits refine more slowly, which may be consistent with our observations of reduced AIS plasticity in S100β-deficient mice. In addition, alterations in optimal S100β levels, through transgenic overexpression, genetic deletion, or pharmacological blockade, have been reported to impair long-term potentiation induction in multiple studies (Lewis and Teyler, 1986; Gerlai et al., 1995; Rebaudo et al., 2000; Nishiyama et al., 2002), supporting the involvement of this glioprotein in activity-dependent plasticity mechanisms requiring precise neuronal timing. Astrocytes also coordinate formation of synaptic circuits in the visual cortex that contribute to CP closure (Ribot et al., 2021), and to modulate synaptic plasticity through release of adenosine and glutamate (Bonansco et al., 2011; Cavaccini et al., 2020; Martínez-Gallego et al., 2022; de Ceglia et al., 2023), and through connexin-30-mediated signaling (Pannasch et al., 2014). Our observations therefore raise the possibility that astrocytes, through astrocyte-derived S100β contributes to mechanisms involved in CP onset. Although the present study does not allow us to discriminate between the intra- and extracellular effects of S100β, this duality of action remains an important consideration for future investigations aimed at elucidating its role during development and upon release.

### S100β deficiency reduces intrinsic excitability of layer V pyramidal neurons

In addition to structural AIS changes, S100β-deficient mice showed reduced intrinsic excitability in L5PNs of the visual cortex, particularly at P21. At this age neurons also exhibited a shorter AIS, which may contribute to the reduced excitability observed, consistent with previous studies where AIS shortening has been associated with decreased neuronal excitability in different neuronal populations (Galliano et al., 2021; Fréal et al., 2023; Jamann et al., 2021). An important function of the S100β protein widely demonstrated is its ability to regulate intrinsic excitability and neuronal firing patterns through reduction of extracellular calcium, both in L5PNs of the visual cortex and in neurons of the mesencephalic trigeminal system (Morquette et al., 2015; Ryczko et al., 2021; Gaudel et al., 2025). Therefore, its absence may contribute to the alterations in intrinsic excitability observed here. Notably, at P7, WT mice displayed greater intrinsic excitability despite possessing a slightly shorter AIS compared to S100β-KO mice. Similarly, at P15, no difference in excitability is observed despite a pronounced difference in AIS length. This indicates that AIS length alone does not fully determine intrinsic excitability, with additional factors such as input resistance, ion channel composition and distribution, or broader developmental processes likely playing a role. A differential ratio of sodium channels NaV1.2 and NaV1.6, which have distinct functions in regulating cortical excitability, may partly account for this dissociation (Garcia et al., 2025). An alternative explanation may involve an accelerated developmental trajectory, whereby S100β-KO mice reach maturation earlier than controls, potentially around P7. One limitation of our study is that, because our model is a constitutive knockout, we cannot determine the cellular source of S100β release responsible for the observed effects. However, S100β is strongly expressed in the central nervous system, primarily by astrocytes but also by maturing oligodendrocytes and certain neuronal populations (Donato et al., 2009; Sorci et al., 2010). Several studies have demonstrated that neuron–oligodendrocyte–astrocyte signaling mechanisms can induce biophysical changes in the axon and modulate neuronal excitability and axonal conduction (Yamazaki et al., 2010; Battefeld et al., 2016; Micu et al., 2016). Additionally, WT and S100β-KO mice differ in their genetic background (B6J vs. B6Brd), and a strain-specific effect cannot be excluded.

## Conclusion

Many functions have been attributed to the calcium-binding protein S100β (Donato et al., 2009), yet its full range of roles has not been completely uncovered. Our results suggest that S100β does not appear to be required for the final AIS state at the peak of the visual CP, but rather for the dynamics of AIS maturation and for early adaptation to sensory activity during the precritical period and the onset of the CP. A better understanding of the mechanisms governing the opening and closure of the visual CP may ultimately help identify approaches to re-engage structural plasticity in the adult brain, with potential therapeutic implications for brain injury and neurodevelopmental disorders.

## Materiel and methods

### Animals

All experiments were conducted in accordance with the rules of the Canadian Institutes of Health Research and were approved by the Animal Care and Use Committee of the University of Montreal (Protocol #23-207). C57BL/6J (WT) and S100β-Knockout (S100β-KO) transgenic mice (B6Brd; B6N-Tyrc-Brd S100btm1a(EUCOMM)Wtsi/WtsiCnbc, S100b (MCFR; EPD0157_6_C08) Wellcome Trust Sanger Institute) were used in this study. WT or S100β-KO were fed *ad libitum* and housed with a 12 h light/dark cycle. Experiments were conducted using both male and female WT and S100β-KO mice. Given the challenges associated with sex determination at early developmental stages, however, we are unable to report the number of male and female animals with confidence.

### Immunohistochemistry and confocal microscopy

For the immunohistochemistry against AnkG, mice were deeply anesthetized with isoflurane and killed by decapitation. Brains were then removed and immersed overnight at 4 °C in 4% (wt/vol) paraformaldehyde (PFA) in PBS. Coronal brain sections (100 μm) were prepared from 7- 15-, 21-, to 28-day-old WT or S100β-KO mice using a vibratome (Vibratome Series 1000, Technical Products International). The sections were rinsed 3 times for 10 min in PBS and incubated in a blocking solution containing 0.3% Triton X-100 and 5% Normal Goat Serum (Jackson ImmunoResearch #017-000-121) in PBS for 1 h at room temperature. The sections were then rinsed 3 times for 10 min in PBS and incubated overnight at 4 °C with the primary antibody (chicken anti-AnkG, EnCor Biotech #PCAC-ANK3, dilution 1:1000). The following day, the sections were rinsed 5 times for 10 min in PBS and incubated with the secondary antibody goat or donkey anti-chicken IgY (H+L) Alexa Fluor 488 (Invitrogen, Thermo Fisher Scientific #A11039, dilution 1:250) diluted in the blocking solution for 120 min in a dark chamber at room temperature. The sections were then rinsed 4 times for 15 min in PBS and mounted on ColorFrost Plus slides (Fisher Scientific, Ottawa, Ontario, Canada) using Fluoromount-G (Southern Biotech, Birmingham, Alabama, USA). Imaging was performed using a Zeiss LSM-900 Confocal Airyscan microscope (Zeiss).

### Image acquisition and processing

AIS analysis and measurements (in pixels) were performed in a blinded manner by multiple experimenters, with mouse identity and experimental day concealed during quantification using ImageJ and the neurite tracer plugin. Only AIS entirely contained within a single optical plane (i.e., a single z-stack slice) were included. Z-stacks were acquired at a resolution of 512 × 512 pixels with 1 μm step sizes using a 40× objective. Confocal images were post-processed and analyzed using ImageJ (version 2.0).

### Electrophysiology

Visual Cortex *ex vivo* slices were obtained from P7, P15, P21 or P28 old WT or S100β-KO mice. Mice were deeply anesthetized with isoflurane and killed by decapitation. Coronal Slices (350 μm) were cut on a vibratome (Leica Microsystems, VT1000S) in an ice-cold (0–4 °C) sucrose-based solution containing the following (in mM): 219 sucrose, 26 NaHCO3, 10 dextrose, 3 KCl, 0.2 CaCl2, 1.25 KH2PO4 and 4 MgSO4 (pH 7.3–7.4, 300–320 mOsmol/kg) and were transferred at 32 °C in regular ACSF containing the following (in mM): 124 NaCl, 3 KCl, 1.3 MgSO4, 26 NaHCO3, 1.2 CaCl2, 1.25 KH2PO4 and 10 dextrose saturated with 95% O2/5% CO2 (pH 7.3–7.4, 300–320 mOsmol/kg) for 15 min before resting at room temperature (RT) for 1 h in oxygenated (95% O2/5% CO2) ACSF. Recordings were obtained using a Multiclamp 700B (Molecular Devices) amplifier and pClamp10.4 software. Data were sampled at 33 kHz, filtered at 3 kHz, and digitized by a Digidata 1440A (Molecular Devices). All data analyses were performed with Clampfit (Molecular Devices). Access resistance was monitored throughout the recording and only experiments with stable resistance were kept (changes < 20%). Recordings were made from layer V pyramidal neurons; electrodes were filled with a solution containing the following (in mM): 140 K-gluconate, 5 NaCl, 20 KCl, 10 HEPES, 2 MgCl2, 0.5 EGTA, 0.4 Tris-GTP and 2 Tris-ATP. All experiments were performed in presence of synaptic blockers: D,L-2-amino-5-phosphonovaleric acid (APV, 75 μM), CNQX (10 μM), SR 95531 hydrobromide (Gabazine, 20 μM). Chemicals used in this study were purchased from Sigma-Aldrich (Oakville, Ontario, Canada) or Tocris Biosciences (Ellisville, Missouri, USA).

### Data and statical analysis

Data are presented as mean ± standard error to the mean (SEM) or standard deviation (SD). Statistical analyses were performed depending on the experimental design. Comparisons between independent groups, for which normality could be assumed (Shapiro–Wilk test), were performed using *t*-tests. Input-output curves were analyzed using repeated measures two-way ANOVA (two-way RM ANOVA) with Greenhouse-Geisser correction and Bonferroni *post hoc* testing. Statistical significance was set at *P < 0.05, **P < 0.01, ***P < 0.001, and ****P < 0.0001; NS indicates not significant. Data analysis was performed using SPSS (IBM SPSS Statistics). Rheobase was determined from the input–output curve as the minimal current required to evoke an action potential. Spike threshold was measured using the first-derivative method, defined as the membrane potential at which dV/dt reached a criterion value corresponding to three times the standard deviation of the baseline.

## Data Availability

The original contributions presented in this study are included in this article/supplementary material, further inquiries can be directed to the corresponding authors.
